# Co-existing intracranial and extracranial carotid artery atherosclerotic plaques and recurrent stroke risk: a three-dimensional multicontrast cardiovascular magnetic resonance study

**DOI:** 10.1186/s12968-016-0309-3

**Published:** 2016-12-02

**Authors:** Yilan Xu, Chun Yuan, Zechen Zhou, Le He, Donghua Mi, Rui Li, Yuanyuan Cui, Yilong Wang, Yongjun Wang, Gaifen Liu, Zhuozhao Zheng, Xihai Zhao

**Affiliations:** 1 Department of Radiology, Beijing Tsinghua Changgung Hospital, Tsinghua University, Beijing, China; 2 Center for Biomedical Imaging Research, Department of Biomedical Engineering, Tsinghua University School of Medicine, Beijing, China; 3 Department of Radiology, University of Washington, Seattle, WA USA; 4 Department of Neurology, Beijing Tiantan Hospital, Capital Medical University, Beijing, China; 5 Department of Radiology, PLA General Hospital, Beijing, China; 6 Center of Stroke, Beijing Institute for Brain Disorders, Beijing, China; 7 Center for Biomedical Imaging Research, Tsinghua University School of Medicine, Haidian District, 100084 Beijing, China

**Keywords:** Intracranial artery, Carotid artery, Atherosclerosis, Cardiovascular magnetic resonance, Recurrent stroke

## Abstract

**Background:**

As a systemic disease, atherosclerosis commonly affects intracranial and extracranial carotid arteries simultaneously which is defined as co-existing plaques. Previous studies demonstrated that co-existing atherosclerotic diseases are significantly associated with ischemic cerebrovascular events. The aim of this study was to investigate the characteristics of co-existing intracranial and extracranial carotid atherosclerotic plaques and their relationships with recurrent stroke by using 3D multi-contrast magnetic resonance (MR) vessel wall imaging.

**Methods:**

Patients with recent cerebrovascular symptoms in anterior circulation and at least one carotid plaque were recruited. All patients underwent cardiovascular magnetic resonance (CMR) for brain and intracranial and extracranial arteries. Presence/absence of atherosclerotic plaque at each arterial segment was identified. The maximum wall thickness (Max WT), length, stenosis of each plaque was measured. The presence/absence of calcification, lipid-rich necrotic core (LRNC), and intraplaque hemorrhage (IPH) was assessed. Cerebral old and acute infarcts in anterior circulation were evaluated.

**Results:**

Fifty-eight patients (mean age: 58.0 ± 8.5 years old, 34 males) were recruited. Of the 58 patients, co-existing intracranial and extracranial carotid artery plaques were found in 45 patients (77.6%), of which 7 (15.6%) had first time acute stroke and 26 (57.8%) had recurrent stroke. For these 33 patients with stroke, the number of intracranial plaques (OR = 11.26; 95% CI, 1.27–100; *p* = 0.030) and co-existing intracranial and extracranial carotid artery plaques (OR = 2.42; 95% CI, 1.04–5.64; *p* = 0.040) was significantly associated with recurrent stroke. After adjusting for traditional risk factors, the number of co-existing plaques was still significantly correlated with recurrent stroke (OR = 3.31; 95% CI, 1.09–10.08; *p* = 0.035). No correlations were found between recurrent stroke and Max WT, length, stenosis, and compositions of plaques.

**Conclusions:**

Co-existing intracranial and extracranial carotid artery plaques are prevalent in symptomatic patients and the number of co-existing plaques is independently associated with the risk of recurrent stroke.

## Background

Cerebrovascular atherosclerotic disease is the major cause of ischemic stroke or transient ischemia attack (TIA) [1, 2]. As a systemic disease, atherosclerosis commonly affects intracranial and extracranial carotid arteries simultaneously which is defined as co-existing lesions [3, 4]. Previous studies demonstrated that co-existing atherosclerotic diseases are significantly associated with ischemic cerebrovascular events [5, 6]. As such, accurate identification of co-existing plaques is important for stroke prevention.

Currently, noninvasive angiography-based imaging techniques, such as computed tomography angiography (CTA) and cardiovascular magnetic resonance (CMR) angiography have been largely utilized for assessment of cerebrovascular atherosclerotic disease severity. However, increased evidences showed that only measuring luminal stenosis by angiography will underestimate the severity of atherosclerotic disease due to the arterial positive remodeling effect [7]. Investigators suggested direct evaluation of the plaque compositional features in the arterial wall especially for arteries with lower grade stenosis [8, 9].

Vessel wall CMR has been demonstrated to be capable of determining atherosclerotic plaque vulnerability in extracranial carotid arteries [10]. In contrast, the validation of in vivo intracranial vessel wall CMR is challenging due to the availability of plaque specimen in living patients. Recent studies validated the performance of ex vivo CMR in evaluation of intracranial intraplaque hemorrhage compared with histology [11]. Yang et al. [12] have shown that the intra- and inter-observer reproducibility of vessel wall CMR in identifying intracranial artery plaque compositions was acceptable. In addition, quantitative CMR, such as T2 mapping, has been successfully utilized to characterize the carotid plaque types [13]. However, most of previous studies were conducted by using two dimensional (2D) imaging techniques. Recently, investigators proposed 3D imaging sequences with large longitudinal coverage and isotropic high spatial resolution which have the potential to characterize atherosclerotic diseases in both intracranial and extracranial carotid arteries in a single scan [14–19].

In this study, we sought to investigate the characteristics of co-existing atherosclerotic plaques in intracranial and extracranial carotid arteries and their relationships with recurrent stroke risk by using 3D multicontrast vessel CMR.

## Methods

### Study sample

Patients with clinically confirmed recent ischemic stroke or TIA in the anterior circulation (<2 weeks) and atherosclerotic plaque in at least one carotid artery determined by ultrasound were consecutively recruited in this study. All subjects underwent MR imaging for brain and intracranial and extracranial carotid arteries. The exclusion criteria include: 1) cardiogenic stroke; 2) hemorrhagic stroke; 3) stroke in posterior circulation; 4) contraindications to CMR. The clinical characteristics including age, gender, body mass index (BMI), history of hypertension, diabetes mellitus, smoking, hyperlipidemia, stroke, TIA, and coronary heart disease and the levels for cholesterol and the blood pressure were collected from the clinical record. The study protocol was approved by institutional review board and the written consent forms were obtained from all the subjects prior to the initiation of this study.

### CMR

CMR was performed on a 3 T MR scanner (Philips Achieva TX, Philips Healthcare, The Netherlands) with a custom-designed 36-channel neurovascular coil [18]. For intracranial and extracranial carotid artery vessel wall imaging, a multicontrast 3D vessel wall imaging protocol was conducted to acquire MERGE, SNAP and T2-VISTA sequences [18] with the following parameters: MERGE: fast field echo (FFE), repeat time (TR)/ echo time (TE) 9.2/4.3 ms, flip angle 6°; SNAP: FFE, TR/TE 9.9/4.8 ms, flip angle 11/5°; and T2-VISTA: turbo spin echo (TSE), TR/TE 2500/278 ms, flip angle 90°. All 3D imaging sequences were acquired coronally with the same field of view of 40 × 160 × 250 mm^3^ and isotropic spatial resolution of 0.8 × 0.8 × 0.8 mm^3^. Gadolinium contrast media was not applied to the vessel wall CMR. A clinically standard imaging protocol including T1-weighted (T1W), T2-weighted (T2W) and diffusion weighted imaging (DWI) was used to acquire the axial images of brain.

### Image review

Four radiologists with >5 year experience in neurological radiology independently interpreted the CMR images with consensus using MR workstation (Philips Extended MR WorkSpace 2.6.3.4, Best, The Netherlands). The intracranial and extracranial carotid arteries were divided into the following segments: M1 segment of middle cerebral artery (MCA), C3-7 segment of internal carotid artery (ICA), C2 segment of ICA, C1 segment of ICA, bulb, and common carotid artery (CCA). The vascular CMR images were reviewed by two radiologists (Y.X. and X.Z.) blinded to brain images and clinical information. Presence or absence of atherosclerotic plaque which is defined as the eccentric thickening of vessel wall at each arterial segment was identified. The maximum wall thickness (Max WT), length, stenosis of each plaque was measured. The luminal stenosis was measured by using the NASCET [20] and WASID [21] criteria for carotid artery and intracranial arteries, respectively. The presence or absence of plaque compositions such as calcification, LRNC, and IPH was assessed for each plaque with the published criteria [10, 15]. The criteria for identification of plaque compositions were as follows: calcification: hypointense on all image sequences; LRNC: isointense or slight hypointense on MERGE images with T1W and hypointense on T2-VISTA images; IPH: hyperintense on SNAP images. The brain MR images were reviewed by the other two radiologists (L.H. and Y.C.) blinded to vascular images and clinical information. The acute infarcts were defined as lesions with hyperintense on DWI and isointense on T1W images. In contrast, the old infarcts were defined as lesions with hypointense on T1W and hyperintense on T2W images.

### Reproducibility study

Ten subjects were randomly selected to determine the intra-observer and inter-observer reproducibility in identification of the presence of plaque and the plaque compositions, including calcification, LRNC, and IPH. The reproducibility for the measurements of Max WT, length, and stenosis of each plaque was also evaluated. A time interval of 3 months was set for testing the intra-observer reproducibility to minimize the bias of memory.

### Statistical analysis

The continuous variables were described as mean value and the corresponding standard deviation (SD) and the binary variables were presented as frequency. The prevalences of atherosclerotic plaque and plaque compositions in intracranial arteries, extracranial carotid arteries and co-existing plaques were determined. Logistic regression was utilized to determine the odds ratio (OR) and corresponding 95% confidence interval (CI) of plaque characteristics in discriminating presence of recurrent stroke in patients with co-existing intracranial and extracranial carotid artery plaques. Cohen’s kappa was calculated to determine the inter-observer and intra-observer agreement in identification of arterial plaque. The intra-class correlation coefficient (ICC) was analyzed to assess inter-observer and intra-observer agreements in quantitatively measuring the atherosclerotic plaque. A *p* value of *p* <0.05 was considered as statistically significant. All statistical analyses were performed using SPSS 16.0 (SSPS Inc. Chicago, IL, USA).

## Results

In total, 64 patients were recruited for CMR. Of 64 patients, 6 were excluded from statistical analysis due to overall poor image quality. Of 58 patients (mean age, 58 ± 8.5 years old) who were included in the final analysis, 34 are males, 40 (69%) had hypertension, 21 (36.2%) had diabetes, 49 (84.5%) had hyperlipidemia, and 30 (51.7%) had history of smoking. The clinical characteristics of this study population are summarized in Table 1. The prevalence of hyperlipidemia of patients with co-existing intracranial and extracranial carotid artery plaques was significantly greater than that of patients with only extracranial carotid artery plaques (91.1% vs 61.5%, *p* = 0.009). No significant differences can be observed in other clinical characteristics between these two patient groups (Table 1). Of 58 patients, 9 (15.6%) had first time acute stroke, 34 (58.6%) had recurrent stroke in the territory of anterior circulation, 10 (17.2%) had only old infarcts, and no infarcts were evidenced on MR images in 5 (8.6%) patients.

**Table 1:** Summary of clinical characteristics of this study population (*N* = 58)

	Mean ± SD or *N* (%)			*p*
	All patients	Patients with only carotid plaques	Patients with co-existing plaques	
Age, years	58.0 ± 8.5	57.2 ± 9.0	58.3 ± 8.5	0.703
Sex, male	34 (58.6)	4 (30.8)	30 (66.7)	0.021
BMI, kg/m^2^	25.5 ± 3.4	27.0 ± 3.1	25.0 ± 3.4	0.065
SBP, mmHg	142.1 ± 24.7	141.9 ± 23.8	142.2 ± 25.2	0.970
DBP, mmHg	90.2 ± 13.9	89.6 ± 12.5	90.3 ± 14.4	0.871
Hypertension	40 (69)	9 (69.2)	31 (68.9)	0.981
Diabetes	21 (36.2)	4 (30.8)	17 (37.8)	0.643
Smoking	30 (51.7)	5 (38.5)	25 (55.6)	0.277
Hyperlipidemia	49 (84.5)	8 (61.5)	41 (91.1)	0.009
Statin	45 (77.6)	8 (61.5)	37 (82.2)	0.115
LDL, mmol/L	3.1 ± 1.2	2.6 ± 0.6	3.3 ± 1.2	0.081
HDL, mmol/L	1.1 ± 0.4	1.1 ± 0.3	1.1 ± 0.4	0.759
TC, mmol/L	4.7 ± 1.0	4.4 ± 0.9	4.8 ± 1.0	0.154
TG, mmol/L	1.7 ± 0.8	1.6 ± 0.7	1.7 ± 0.8	0.745
Stroke	52 (89.7)	12 (92.3)	40 (88.9)	0.721
TIA	18 (31.0)	4 (30.8)	14 (31.1)	0.981
Coronary heart disease	10 (17.2)	2 (15.4)	8 (17.8)	0.841

*Abbreviations*: *BMI* body mass index, *LDL* lower density lipoprotein, *HDL* high density lipoprotein, *TC* total cholesterol, *TG* triglycerides

### Characteristics of intracranial and extracranial carotid artery plaques

**Fig. 1:**
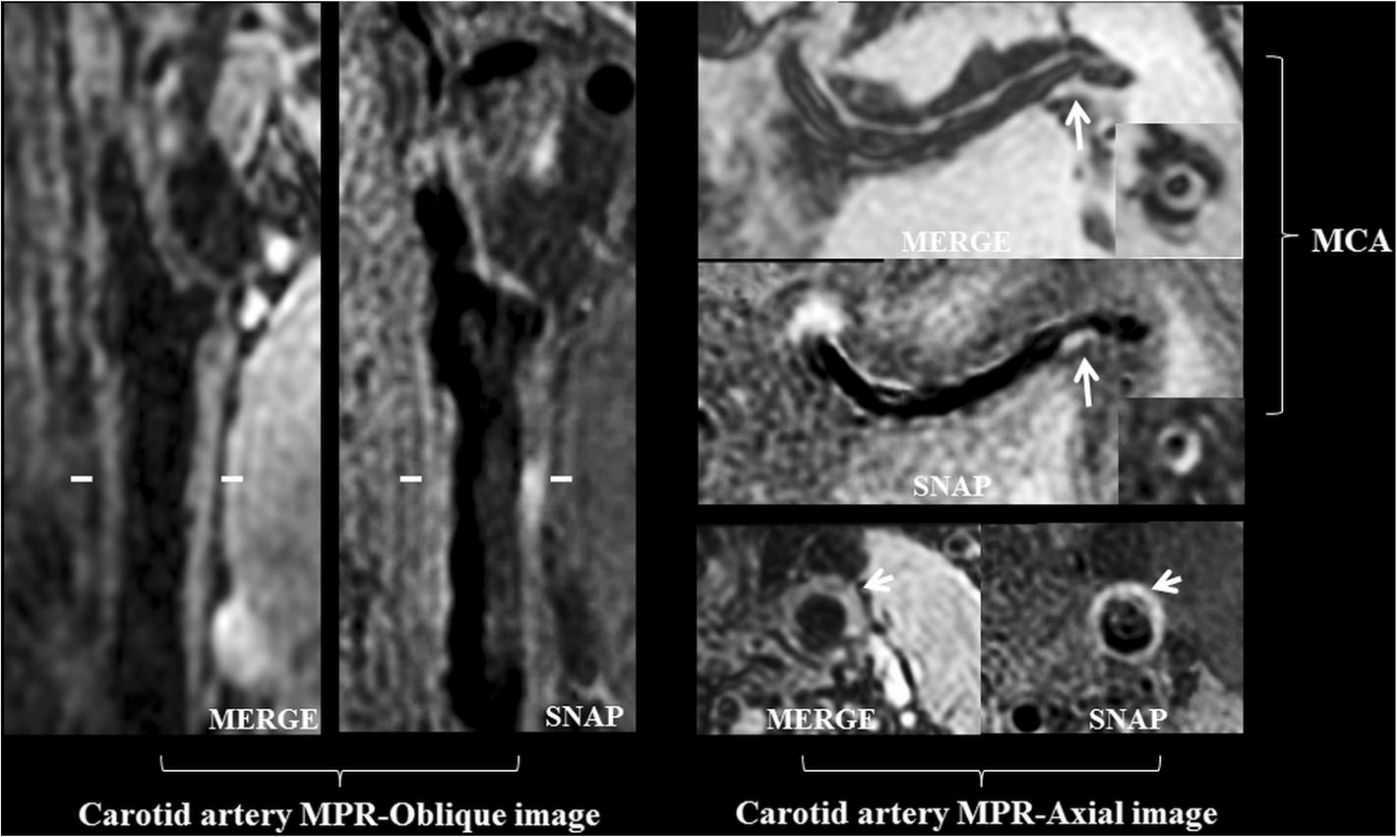
One patient with co-existing intracranial and extracranial carotid artery atherosclerotic plaques. In the left common carotid artery (CCA), an eccentric plaque was found on multiple planar reconstructed (MPR)-oblique and MPR-axial MERGE and SNAP images (arrows). The plaque in left CCA showed hyperintense on SNAP image, indicating presence of IPH. In the same patient, an eccentric plaque was found in the left middle cerebral artery (MCA) on curved reconstructed MERGE and SNAP images (arrows) and corresponding MPR-axial images (lower right corner). The plaque in left MCA appeared hyperintense on SNAP image, indicating presence of IPH

In total, 209 atherosclerotic plaques were detected in all 58 patients. Of 209 plaques detected, 114 (54.5%) were found in extracranial carotid arteries and 95 (45.5%) occurred in intracranial arteries. Co-existing intracranial and extracranial carotid artery plaques were found in 45 patients (77.6%). Figure 1 showed an example of co-existing intracranial and extracranial carotid artery plaques in one patient and both atherosclerotic plaques appeared hyperintense on SNAP images indicating high risk lesions. Table 2 summarized the burden and compositional features of intracranial and extracranial carotid artery plaques. For intracranial artery atherosclerotic plaques, the mean stenosis of all lesions was 27.6% ± 13.9%, and the prevalence of calcification, LRNC, and IPH was 17.9, 45.3, and 15.8%, respectively. For the extracranial carotid artery plaques, the stenosis was 19.4% ± 17.3%, and the prevalence of calcification, LRNC, and IPH was 26.3, 70.2, and 15.8%, respectively.

**Table 2:** Characteristics of intracranial and extracranial plaques on MR images

	Mean ± SD or *N* (%)	Range
Intracranial artery		
Maximum wall thickness, mm	2.3 ± 0.7	1.1–3.5
Length, mm	8.6 ± 3.6	2.9–18.5
Stenosis, %	27.6 ± 13.9	2.2–68.3
Presence of calcification	17 (17.9)	
Presence of lipid-rich necrotic core	43 (45.3)	
Presence of intraplaque hemorrhage	15 (15.8)	
Extracranial carotid artery		
Maximum wall thickness, mm	3.6 ± 1.1	2.2–8.0
Length, mm	13.2 ± 6.1	4.5–31.8
Stenosis, %	19.4 ± 17.3	0–100
Presence of calcification	30 (26.3)	
Presence of lipid-rich necrotic core	80 (70.2)	
Presence of intraplaque hemorrhage	18 (15.8)	

### Correlation between plaque characteristics and recurrent stroke

**Fig. 2:**
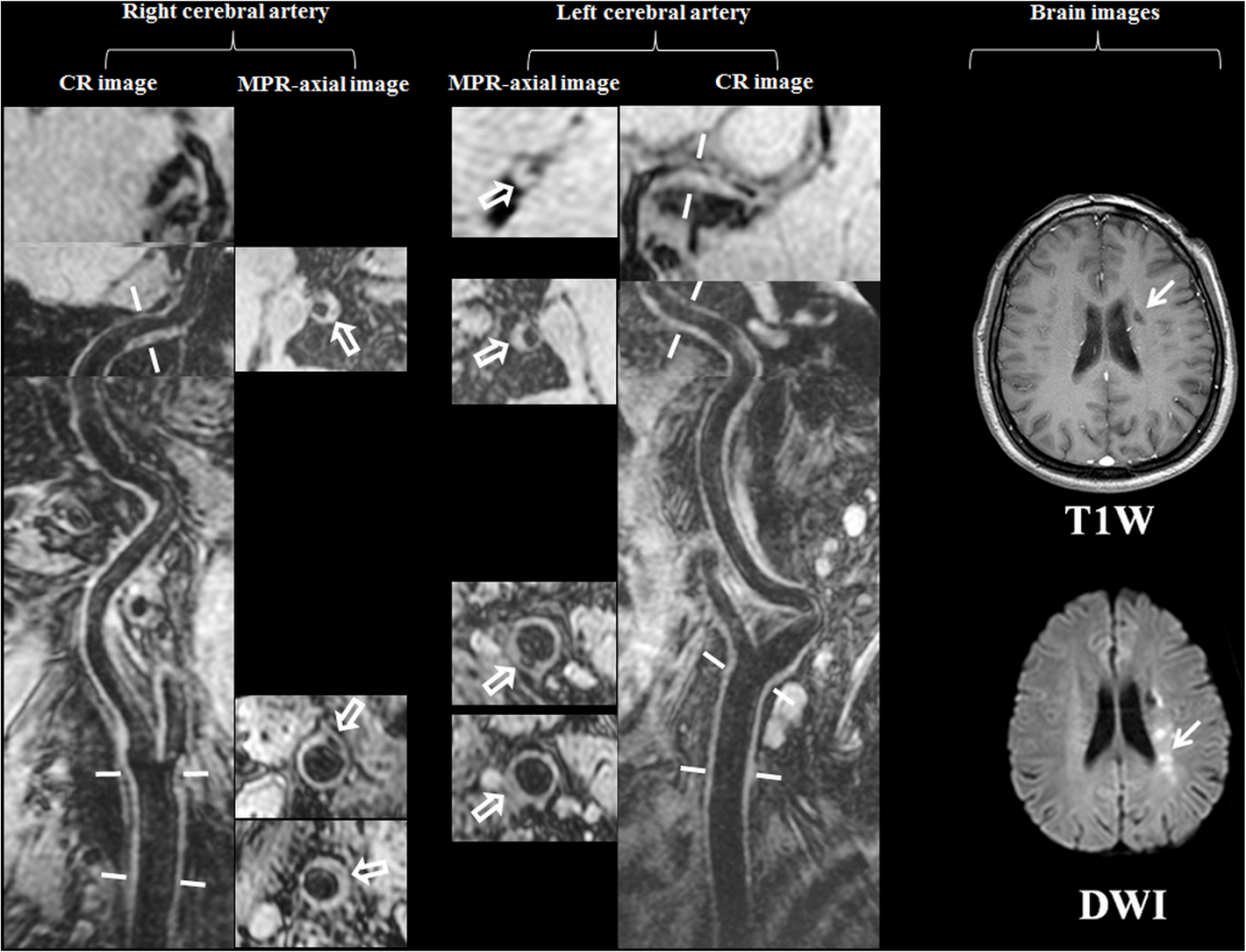
One patient with multiple co-existing intra- and extra-cranial plaques and recurrent stroke. The old infarct showed hypointense on brain T1W image (arrow) and the acute infarct showed hyperintense on brain DWI image (arrow). The multiple plaques can be seen on both left and right curved reconstructed (CR) images of MERGE. Seven plaques (hollow arrows) were identified on axial images after multiple planner reconstruction (MPR) in different cerebral artery segments

Of 45 patients with co-existing intracranial and extracranial carotid artery plaques, 7 (15.6%) had first time acute stroke, 26 (57.8%) had recurrent stroke in the territory of anterior circulation, 8 (17.8%) had only old infarcts, and no infarcts were evidenced on MR images in 4 (8.9%) patients. For the 33 patients with co-existing intracranial and extracranial carotid artery plaques and either first time acute stroke or recurrent stroke in anterior circulation, the number of intracranial plaques (OR = 11.26; 95% CI, 1.27–100.23; *p* = 0.030) and the number of co-existing intracranial and extracranial carotid artery plaques (OR = 2.42; 95% CI, 1.04–5.64; *p* = 0.040) were found to be significantly associated with recurrent stroke. After adjusting for traditional risk factors, including age, gender, BMI, history of hypertension, smoking, diabetes, and hyperlipidemia, and the recurrent stroke was still significantly correlated with the number of co-existing intracranial and extracranial carotid artery plaques (OR = 3.16; 95% CI, 1.03–9.71; *p* = 0.044) but not with the number of intracranial artery plaques (OR = 15.93; 95% CI, 0.92–275.99; *p* = 0.057). No significant correlations were found between recurrent stroke and Max WT, length, and stenosis of plaques (Table 3). Figure 2 was an example showing multiple atherosclerotic plaques in both intracranial and extracranial carotid arteries and cerebral old and acute infarcts.

**Table 3:** Association between plaque characteristics and recurrent stroke

	Discriminating presence of recurrent stroke		
	OR	95% CI	*p*
Before adjustment^a^			
Intracranial plaques			
MaxWT, mm	1.06	0.30–3.670	0.929
Length	1.02	0.74–1.42	0.887
Stenosis	1.03	0.95–1.12	0.478
Calcification	1.05	0.230–3.75	0.939
LRNC	1.67	0.52–5.43	0.393
IPH	2.17	0.32–14.85	0.429
Plaque number	11.26	1.27–100.23	0.030
Extracranial plaques			
MaxWT	2.01	0.58–7.01	0.274
Length	1.08	0.90–1.31	0.406
Stenosis	0.98	0.92–1.03	0.374
Calcification	2.80	0.39–20.17	0.307
LRNC	1.01	0.40–2.60	0.977
Plaque number	1.39	0.49–3.98	0.536
Co-existing plaques			
Plaque number	2.42	1.04–5.64	0.040
After adjustment^a^			
Intracranial plaques			
MaxWT, mm	0.80	0.20–3.24	0.755
Length	0.89	0.60–1.33	0.573
Stenosis	1.06	0.96–1.18	0.221
Calcification	0.49	0.07–3.41	0.469
LRNC	1.41	0.36–5.48	0.621
IPH	1.00	0.05–21.91	0.999
Plaque number	15.93	0.92–275.99	0.057
Extracranial plaques			
MaxWT	3.05	0.57–16.34	0.193
Length	1.14	0.871.50	0.342
Stenosis	0.98	0.92–1.04	0.515
Calcification	3.77	0.40–35.75	0.248
LRNC	1.05	0.29–3.77	0.940
Plaque number	1.42	0.44–4.59	0.562
Co-existing plaques			
Plaque number	3.16	1.03–9.71	0.044

^a^The logistic regression was conducted before and after adjusting for the traditional risk factors, including age, gender, BMI, history of hypertension, smoke, diabetes, and hyperlipidemia

### Reproducibility

For intra-observer agreement in identification of presence of atherosclerotic plaque, the kappa value was 1, 0.89, 0.88, 1, 0.83 and 0.77 for CCA, bulb, C1, C2, C3-7, and M1 segment, respectively (all *p* < 0.001). For the intra-observer agreement in assessment of presence of plaque compositions, the kappa value was 0.85, 0.74, and 0.71 for calcification, LRNC, and IPH, respectively (all *p* < 0.001). The intra-observer ICC was 0.98 (95% CI, 0.96–0.99), 1.00 (95% CI, 0.96–1.00), and 0.96 (95% CI, 0.92–0.98) for measuring Max WT, length, and stenosis, respectively.

For inter-observer agreement in identification of presence of atherosclerotic plaque, the kappa value was 1, 0.89, 0.76, 1, 0.61 and 0.62 for CCA, bulb, C1, C2, C3-7, and M1 segment, respectively (all *p* < 0.01). For the inter-observer agreement in assessment of presence of plaque compositions, the kappa value was 0.66, 0.69, and 0.67 for calcification, LRNC, and IPH, respectively (all *p* < 0.001). The inter-observer ICC was 0.95 (95% CI, 0.89–0.97), 0.95 (95% CI, 0.91–0.98), and 0.91 (95% CI, 0.81–0.96) for measuring Max WT, length, and stenosis, respectively.

## Discussion

This study investigated the characteristics of co-existing intracranial and extracranial artery plaques and their relationships with recurrent stroke by using 3D multicontrast vessel wall CMR. We found that more than 3/4 symptomatic patients with carotid artery plaques had co-existing intracranial artery plaques. In patients with co-existing plaques, the number of co-existing plaques was found to be significantly associated with recurrent stroke before and after adjusting for traditional risk factors, suggesting that the number of co-existing intracranial and extracranial carotid artery plaques might be an independent indicator for recurrent stroke risk.

In this study, 77.6% of symptomatic patients with extracranial carotid artery plaques were found to have co-existing intracranial artery plaques. High incidence of co-existing intracranial and extracranial carotid artery plaques was reported ranging from 42.2 to 64% in symptomatic patients in previous studies [3, 4, 22]. Compared with previous studies, the presence of atherosclerotic plaque in either intracranial or extracranial carotid arteries was determined by vessel wall CMR in our study. This imaging technique may yield more lesion detection which may be underestimated by measuring luminal stenosis due to positive remodeling effect [23]. The presence of co-existing plaques further compels the evidence that the atherosclerosis may be developed systemically and both intracranial and extracranial carotid arteries may share the similar risk factors. The high prevalence of co-existing intracranial and extracranial carotid artery plaques in symptomatic patients also suggests that the strategies for diagnosis and treatment may need to target multiple vascular beds once such co-existing lesions present.

In the present study, the number of co-existing intracranial and extracranial plaques was found to be significantly associated with recurrent stroke. Our finding is in line with previous reports. Presence of co-existing plaques in intracranial and extracranial arteries may be an effective indicator for poor prognosis of ischemic stroke patients [5, 6, 24]. Investigators have demonstrated that patients with co-existing plaques have higher risk of future recurrent stroke or death [5, 25]. Previous studies found that the recurrent stroke rate in patients with co-existing plaques was higher than those without co-existing plaques (41% vs 22%, *p* = 0.002) [5]. The underlying mechanisms are thought to be the bigger burden of atherosclerosis and the synergistic effect of concurrent stenosis [5]. Wong et al. [26] found that the number of occlusive arteries was significant predictor for death and future vascular events in stroke patients. Similar results were also seen in patients with some other diseases. Maeda et al. [27] reported that the carotid plaque number measured by ultrasonography is an independent marker for fatal cerebrovascular events in hemodialysis patients. The relationship between plaque number and ischemic events can be also found in coronary arteries. A study by Stolzmann et al. [28] showed that the number of coronary artery plaque determined by CTA is a significant independent predictor for myocardial ischaemia. Sato et al. [29] reported that the number of coronary vulnerable plaques is associated with metabolic syndrome in patients with acute myocardial infarction. The number of atherosclerotic plaques distributed in multiple vascular beds may represent systemic burden of atherosclerotic diseases. Evaluation of co-existing plaque numbers in multiple vascular beds might be important for stratification of cardiovascular disease risk.

In the present study, co-existing intracranial and extracranial artery plaques were more frequently found in men. The male preponderance of co-existing plaques in multiple vessels has been demonstrated in coronary arteries in previous studies. Lansky et al. [30] reported that non culprit lesions were more locally distributed in more vessels in men than women. Morito et al. [31] found that male patients tend to having more extensive plaque burden in multiple vessel plaques. In addition, in this study, significant association can be observed between co-existing plaques and hyperlipidemia. Previous study by Man et al. [32] revealed that genetic polymorphisms affecting lipid metabolism are associated with co-existence of intracranial and extracranial plaques, and thus, are possible risk factors for co-existing plaques.

## Limitations

This study has several limitations. First, this is a cross-sectional study. Prospective studies are warranted to investigate the predictive value of co-existing intracranial and extracranial carotid artery atherosclerosis for future events. Second, we recruited patients with existing carotid plaques. This study population didn’t include those with intracranial atherosclerosis but without extracranial carotid artery plaques. Third, this pilot study has smaller sample size. Future studies with larger sample size are warranted. Finally, 6 patients with poor image quality were excluded from the final analysis. This may introduce the patient selection bias.

## Conclusions

The co-existing intracranial and extracranial carotid artery plaques are prevalent in symptomatic patients and the number of co-existing plaques is independently associated with the risk of recurrent stroke.

## Abbreviations

CCA: Common carotid artery
ICA: Internal carotid artery
IPH: Intraplaque hemorrhage
LRNC: Lipid-rich necrotic core
Max WT: Maximum wall thickness
MCA: Middle cerebral artery
MERGE: Motion sensitized driven Equilibrium prepared Rapid Gradient Echo
SNAP: Simultaneous Non-contrast Angiography and intraPlaque hemorrhage
TIA: Transient ischemia attack
VISTA: Volumetric isotropic turbo spin echo acquisition
